# Undergraduate research- a tale of three African institutions

**DOI:** 10.4314/ahs.v23i2.85

**Published:** 2023-06

**Authors:** Rhena Delport, Abigail Dreyer, Ronel Maart, Asma MohamedSharif, Rebecca Nekaka, Astrid Turner, Jacqueline Wolvaardt

**Affiliations:** 1 Department of Family Medicine, School of Medicine, University of Pretoria, Pretoria, South Africa; 2 Department of Family Medicine and Primary Care, School of Clinical Medicine, University of Witwatersrand, Johannesburg, South Africa; 3 Faculty of Dentistry, University of the Western Cape, South Africa; 4 Department of community medicine faculty of medicine, Al Neelain University, Khartoum, Sudan; 5 Department of Community and Public Health, Busitema University, Uganda; 6 School of Health Systems and Public Health, University of Pretoria, Pretoria, South Africa

**Keywords:** Research, medical students, undergraduate, Africa, competencies

## Abstract

**Background:**

The inclusion of research in undergraduate medical curricula benefits students as well as the scientific community. Multiple studies report the presence of one or more barriers to research training in these curricula.

**Objectives:**

This paper presents and compares three studies done regarding the teaching of research in undergraduate medical curricula in South Africa, Sudan and Uganda.

**Methods:**

Two cross-sectional study designs (South Africa and Sudan) and one interventional study design (Uganda) were conducted. Both cross-sectional studies used mixed methods while the Ugandan study used a quantitative method. A total of 41 faculty members and 554 students participated. The studies used a combination of surveys, focus group discussions, key informant interviews and document analysis.

**Results:**

Participants from all three studies valued research and considered it useful and relevant to their studies. The findings from the South African and Sudanese studies align with the ‘Four I's’ framework that summarise the barriers to research training as lack of initiative, impulse, incentive and idols. The Ugandan study demonstrated improved self-reported knowledge and attitude (specifically anxiety) among participants after completion of a short course on research.

## Introduction

The explosive increase in information, in terms of volume and ease of access, has resulted in a re-imagining of the role of universities and other educational institutions with regard to knowledge generation.^1^ The next generation of health professions students will need to be able to discriminate between vast amounts of information and be able to extract and synthesise knowledge that is necessary for clinical and population-based decision-making.^1^ These competencies align with the trend towards integrating scientific research training into undergraduate medical education 2,3 with critical thinking and critical appraisal skills as two of the expected outcomes of this inclusion.

Understanding research methodology has become an essential pre-requisite for future physicians. 4 Undergraduate research programmes offer benefits to students. For example, the undergraduate research experience is one of several experiences that can affect students' future career choices. The research process also has valuable learning objectives that have a lasting influence as undergraduates prepare for professional service. 5,6 In 2009, a ranking of ten research-related attributes and skills among experts from medicine, dentistry and veterinary science found that the same core attributes and skills are required by those who wish to remain in purely clinical work as well as those who want to do research.^7^ Amongst those attributes and skills were curiosity or an inquiring mind; critical appraisal; evidence base for professional practice; an ability to work in a team and an ability to communicate. Medical students themselves acknowledge the benefits and need for research teaching. 8,9

The integration of the teaching of research in medical education has itself been a topic of inquiry and different models are used to guide academics in conceptualizing undergraduate research opportunities within their context. 10-12

The factors cited as the main barriers to research training in the undergraduate curriculum can be summarized with the use of the ‘Four I's’ framework of Scaria.^13^ This conceptual framework defines the barriers to research training as lack of initiative (exposure, experience and knowledge), impulse (time and competitive environment), incentive (presentation/publication opportunities and acknowledgement) and idols (mentors and supervisors).^13^ All these factors are very real challenges to incorporate research training in medical curricula – especially in resource-constrained settings.^12^,^14^

Conversely, several factors provide a conducive environment for teaching, promoting understanding and appreciation of research that allows for independent research projects. 6,15 These factors include having a mentor 16; attending a course on research projects early in the curriculum 15 which is contextualised 6; utilise elective opportunities for research exposure and teaching 8; and having research expectations that are measurable and appropriately assessed.^6^

According to Kern's model of curriculum design, the needs of the community or society underpin the curriculum development process. 17 Countries classified as low-resource settings usually have rich community-based learning opportunities and research projects that offer students a learning opportunity to identify community needs as well as guide them through the systematic process to establish facts and reach new conclusions. 18

Despite the acknowledgement that the acquisition of research skills during the undergraduate medical curriculum may benefit students, little is known of how best to ensure this inclusion. Medical schools have to weigh the possible benefit of including research skills against the opportunity cost of including additional clinical content. In addition, clinical teachers might not be able, or willing, to teach research skills.

The authors of this paper conducted independent research informed by local needs with regard to the inclusion of research skills in the undergraduate medical curricula in their respective African institutions. This paper therefore presents the findings from three studies done in relation to the teaching of research in undergraduate medical curricula in South Africa, Sudan and Uganda. The background methodology and results of each study is summarised prior to the synthesis in the discussion.

## South Africa

### Background

The undergraduate medical degree in South Africa is intensive, with many discipline-specific subjects jostling for limited time with students over the five- or, more commonly, six-year degree programme. Therefore, the teaching of basic research skills has understandably been restricted, even though the benefits of this teaching are widely acknowledged. 6,19,20

At the University of Pretoria there is a short research module offered in the second-year medical curriculum. This module is the primary research offering within the curriculum, is not credit bearing and has learning outcomes and a format that have changed over time.

The learning objectives of the block are:

- Phrase clear, answerable, relevant questions related to practice.
- Use appropriate techniques to access relevant research findings from reliable sources.
- Conduct a literature review.
- Explain the applicability of research findings.
- Explain the basic principles of research design and analysis as well as research ethics.
- Respect and comply with laws pertaining to plagiarism, confidentiality and ownership of intellectual property when accessing and using information and conducting research.

**Research question:** What factors contribute to generating interest in research among the medical students?

**Research aim:** To explore the factors that generate interest in research among the medical students at the university.

### Methodology

A cross-sectional study design used two student focus group discussions (FGDs) in 2016, three key informant interviews (KIIs) in 2017 and an analysis of end-of-block/module surveys from the module (2015 to 2017). The student participants for the two FGDs were purposively sampled. Due to the nature of the topic, only medical students from second to the final year of study who completed the research module and who had expressed a strong interest or disinterest in research were invited to participate. The participants for the KIIs were purposively sampled based on their involvement in research decision-making at the faculty. Separate FGDs were held based on either interest or disinterest in research (n=7 each). A self-developed focus group discussion guide was used. Anyone who did not meet these inclusion criteria for the FGDs or the KIIs were excluded. Three KIIs of approximately 60 minutes were conducted using self-developed interview guide. The routine data from an end-of-block/module questionnaire were collected via an online Qualtrics survey in 2015 (N=275), 2016 (N=292) and 2017 (N=287) by the Education Office. Anyone who was not enrolled for the module in the years of interest were excluded. From 2016, it consisted of eight closed questions with a Likert scale (1 to 5 from disagree entirely /extremely poor to agree entirely/very good) and one open-ended question.

The audio recordings from the FGDs and KIIs were transcribed and checked for accuracy. The transcripts were manually coded and categorized using a combination of an inductive (derived from the text) and deductive approach (extracted from the literature).

The quantitative data from the surveys were analysed using descriptive statistics in Microsoft Excel 2010. Participants in the FGDs and the KIIs provided written consent to participate. Participation in the FGDs and the KIIs was voluntary and participation was confidential. Participation in the end-of-block questionnaire was voluntary and anonymous. All participants could choose not to participate or to withdraw at any time. Ethical approval for the study was granted (120/2016).

### Findings

The response rate in the online surveys varied over time (51.6% in 2015; 35.6% in 2016 and 19.1% in 2017). In general, the survey respondents rated the perceived contribution of the module in improving research skills and knowledge highly (Table 1).

**Table 1:** The mean (standard deviation, SD) Likert scale responses of students' evaluation of the research module SMO 211 (2015 to 2017)

Survey question/statement	2015 (n=142)	2016 (n=104)	2017 (n=55)
1. The research project improved my research skills regarding a literature review, protocol and research report	3.76 (SD 1.14)	4.02 (SD 0.98)	3.98 (SD 0.98)
2. The research project improved my knowledge about the specific topic	3.36 (SD 1.32)	3.89 (SD 1.08)	3.88 (SD1.12)
3. The workload was too heavy	3.27 (SD 1.29)	2.62 (SD 1.31)	2.42 (SD 1.46)
4. There were sufficient resources and learning materials available for this SMO 211	3.35 (SD 1.29)	3.48 (SD 1.15)	3.73 (SD 1.06)
5. SMO 211 was organised and administered very well	3.08 (SD 1.13)	3.47 (SD 1.26)	2.82 (SD 1.32)
6. My group supervisor communicated effectively with the students	3.79 (SD 1.31)	3.60 (SD 1.32)	3.38 (SD 1.44)
7. The Learning Management System (clickUP) for SMO 211 was useful and easy to navigate	3.30 (SD 1.30)	3.53 (SD 1.24)	3.45 (SD 1.14)
8. SMO 211 exposed students to basic principles of research that they had to apply in the format of a protocol (the first step in the research process)	N/A	3.97 (SD 1.04)	3.96 (SD 0.97)

All participants concurred that the inclusion of research in a second-year module provides a stepping-stone or induction into research. Data triangulation between the FGDs, KIIs and survey analysis highlighted fundamental challenges in the timing, content and duration that affected research interests. Student FGD participants highlighted additional factors that affected their willingness or ability to pursue research further, which included the “fear of doing it on your own”, as well as insufficient, and unknown incentives. Key informants emphasised that role modelling with the presence of a mentor is a critical factor in motivating students to continue further with research. Crucial negative factors related to the research module that emerged from the surveys and FGD participants included the lack of choice among research topics, unwilling and disengaged group members and unreliable and unwilling supervisors.

## Sudan

### Background

The undergraduate medical degree in Sudan is a five-year degree programme. Students at the Al Neelain University attend a course of basic research methodology of three credit hours that equivalent to forty-five notional hours, in their sixth semester (or third year). This course is followed by an individual research project that students complete in semester seven and eight (or fourth year) before submitting a research report.

The learning objectives of the course and the research project are:

- Prepare and submit a research proposal.
- Select, define and state the research problem.
- Write rationale for the research problem.
- Formulate general and specific research objectives.
- Develop and enlist study variables.
- Review and analyse the relevant literature from different sources.
- Summarize and present the literature review using standard frame.
- Use competently the list of variables to develop a questionnaire.
- Develop and pre-test and finalize the data collection instruments.
- Use the main computerized statistical packages and their applications.
- Cite correctly the references in the text and the list.

The quality assurance department of the Al Neelain University expressed concerns regarding the attainment of the expected learning outcomes linked to research in the curriculum. The expected learning outcomes are the development of an inquiring mind, critical appraisal, understanding of the evidence base for professional practice, and an understanding of ethics and governance.

**Research question:** What factors impact on research skills development in undergraduate medical students?

**Research aim:** To explore the development of research skills in the undergraduate medical students at the university.

### Methodology

A cross-sectional study design was used. The registration list from one-year group (n=155) was used to choose a systematic random sample. Nineteen students' research projects reports were evaluated to assess research reporting skills, and the STROBE checklist was adapted and used to evaluate the reports 21. The STROBE checklist 21 outlines the expected inclusions for observational studies and is a common benchmark. In addition, 38 faculty members were purposively sampled and completed an online questionnaire. Anyone who was not a faculty member was excluded. The faculty questionnaires sourced information on the lecturers' specialty, teaching and research experience, number of students they supervise yearly, methods used to assess students' research skills, what they perceived as barriers to student attaining research skills, and what the university could do to motivate them to supervise students effectively. Background information regarding expected learning outcomes, activities and assessment strategies were extracted from curriculum documents (2014-2015). Finally, two annual routine module evaluations that include questions about the students' perceptions of research were used. Participation in the online questionnaire (by faculty) and the annual evaluations (by students) was voluntary and anonymous. Participants could choose not to participate or to withdraw at any time. Ethical approval was granted (NU-IRB-19-3-3-23).

### Findings

Using the adapted STROBE checklist, the mean score for the 19 proposals reviewed was 4.6/10, with the highest score of 5.9/10. Faculty respondents considered the lack of scheduled time for communication between supervisors and students (n=26; 67%), research activities not being well organized (n=17; 45%), and the lack of time to supervise students (n=16; 42%), as the three most important factors affecting skill attainment by students. The poor research skills of faculty (n=4; 10.5%) were also cited as a factor.

The curriculum documents did not mention any activity or assessment linked to the learning outcome related to critical appraisal. Supervisors sign a checklist (primarily a tracking system) when students complete chapters in the report which is assessed orally.

Finally, the module evaluations showed that student respondents (n= 167) reported the lack of competence and commitment of supervisors (n=90; 55%), the insufficient time allocation (n=84; 52%), and the lack of research funds (n=60; 37%) as the three main barriers to perform research.

## Uganda

### Background

The undergraduate medical degree in Uganda is a six-year degree programme. One of the objectives of the Faculty of Health Sciences at Busitema University (a newly established medical school in Uganda) is to establish a strong research partnership with rural primary health care facilities to generate an evidence base to solve community health problems. Health science students spend four weeks at the end of every academic year for three years at a rural health facility. Students learn about community health, environmental sanitation, socio-economic conditions and the health problems of the community during this community placement. There is no formal module regarding research methods in either the nursing or medical curriculum and students' attitudes and knowledge about research is unknown.

**Research question:** What is the knowledge and attitude of undergraduate medical students towards research after a one-week training module in research methods?

**Research aim:** To determine the knowledge and attitudes of undergraduate medical students towards research after a one-week training module in research methods.

### Methodology

An interventional study was carried out among 75 second-year undergraduate medical and nursing students from June to August 2018. No sampling was done as all of those who attended the training was invited to participate. Any medical or nursing student who was not in their second-year was excluded. A self-developed pre-test structured questionnaire that explored knowledge and attitudes towards research was administered. The intervention consisted of one-week of didactic lectures in research methods and hands-on application to community settings.

The outcomes of the short course were:

- Phrase a clear, answerable, relevant research question related to a PHC gap in practice.
- Locate, critically evaluate and interpret relevant & previous research findings from robust sources.
- Consider the applicability of research, understand research design, analysis, and research ethics.
- Consider patient autonomy, respect, plagiarism, confidentiality and ownership of intellectual property.
- Create, apply, translate and disseminate knowledge.

A post-test was conducted after one month of community placement using the same questionnaire. Participation in the pre- and post-test questionnaire was voluntary and anonymous. Participants could choose not to participate or to withdraw at any time. Ethical approval was granted (MRRH-REC OUT 070/2018).

### Findings

Almost all the students participated (96%; n=72). The majority of those who participated (83%; n=60) were second-year medical students. The sample consisted of almost equal representation of male (50.4%) and female (49.6%) students. The mean age of the participants was 24 (SD: 4.5) years. The median age was 23 years with an interquartile range of 21-28 years.

The knowledge scores were dichotomized based on the median value of pre-test scores. The median knowledge score during the pre-test was 67%, which increased to 73% in the post-test assessment (p< 0.0011) (Table 2).

**Table 2:** Participants' knowledge scores

Knowledge scores	Pre-test	Post test
Median	67(60-73)	73(67-80)
Mean	64.9	72

Total	72	72

*Statistically significant – Wilcoxon sign rank test P<0.0011.

Most of the participants considered research useful and relevant to their studies prior to the intervention (Table 2). Attitudes towards research shifted positively after the research module. Research anxiety was measured as a combination of seven statements related to stress and tension (Table 3). The post-test results showed no significant change in research anxiety. A positive attitude towards research (seven statements) and relevance of research to life (five statements) showed no significant change after the intervention. Research difficulty was measured as a combination of two statements but although the perception of difficulty was lower after the intervention, the difference was borderline (p=0.0705).

**Table 3:** Attitudes towards research pre- and post-attendance of a short course in research methods

VARIABLE	PRETEST			POST TEST		
Research Usefulness	Agree	neutral	Disagree	Agree	Neutral	Disagree
I think medical research is a good career for me	65(90.3)	5(7)	2(2.8)	65(90.3)	4(5.6)	3(4.2)
Research training should be a part of undergraduate curriculum	71(98.6)	0(0)	1(1.4)	71(98.6)	0(0)	1(1.4)
Research should be incorporated in professional training	69(95.8)	2(2.8)	1(1.4)	68(94.4)	1(1.4)	3(4.2)
Performing research is useful and valuable for my profession	71(98.6)	1(1.4)	0(0)	72(100)	0(0)	0(0)
The stills acquired in research will be helpful to me in future	69(95.8)	1(1.4)	2(2.8)	71(98.6)	1(1.4)	0(0)
Research is important to advance knowledge	70(97.2)	2(2.8)	0(0)	71(98.6)	1(1.4)	0(0)
Research should be taught to all students	62(86.1)	5(6.9)	5(6.9)	58(80.6)	9(12.5)	5(6.9)
Research is useful to every professional	62(86.1)	9(12.5)	1(1.4)	59(81.9)	8(11.1)	5(6.9)
I will employ research approaches in my profession	62(86.1)	9(12.5)	1(1.4)	62(86.1)	7(9.7)	3(4.2)
						
**Research Anxiety**						
Research makes me nervous	26(36.1)	7(9.7)	39(54.2)	21(29.2)	16(22.2)	35(48.6)
Performing research is stressful	54(75)	9(12.5)	9(12.5)	41(56.9)	12(16.7)	19(26.4)
Research makes me anxious	31(43.1)	13(18.1)	28(38.9)	19(26.4)	14(19.4)	39(54.2)
Research scares me	15(20.8)	11(15.3)	46(63.9)	10(13.9)	10(13.9)	52(72.2)
Research is complicated	21(29.2)	9(12.5)	42(58.3)	21(29.2)	13(18.1)	38(52.8)
Performing research is a complex subject	38(52.8)	8(11.1)	26(36.1)	29(40.3)	7(9.7)	36(50.0)
I feel insecure concerning the analysis of research data	16(22.2)	13(18.1)	43(59.7)	18(25)	6(8.3)	48(66.7)
Research is difficult	15(20.8)	16(22.2)	41(56.9)	16(22.2)	8(11.1)	48(66.7)
						
**Positive Attitude towards Research**						
I love research	63(87.5)	4(5.6)	5(6.9)	64(88.9)	3(4.2)	5(6.9)
I enjoy performing research	61(84.7)	7(9.7)	4(5.6)	61(84.7)	4(5.6)	7(9.7)
Most students benefit from research	64(88.9)	7(9.7)	1(1.4)	62(86.1)	8(11.1)	2(2.8)
I am interested in research	65(90.3)	4(5.6)	3(4.2)	62(86.1)	5(6.9)	5(6.9)
Research is interesting	60(83.3)	10(13.9)	2(2.8)	63(87.5)	8(11.1)	1(1.4)
Research acquired knowledge is as useful as arithmetic	57(79.2)	9(12.5)	6(8.3)	59(81.9)	11(15.3)	2(2.8)
I am inclined to study the details of research	55(76.4)	13(18.1)	4(5.6)	58(80.6)	12(16.7)	2(2.8)
**RESEARCH RELEVANCY TO LIFE**						
Research-orientated thinking plays an important role in everyday life	66(91.7)	4(5.6)	2(2.8)	68(94.4)	3(4.2)	1(1.4)
Research is important to discover new things	70(97.2)	2(2.8)	0(0)	71(98.6)	1(1.4)	0(0)
I use research in my daily life	37(51.4)	20(27.8)	15(20.8)	40(55.6)	15(20.8)	17(23.6)
Research thinking does not apply to my personal life	13(18.1)	10(13.9)	49(68.1)	11(15.3)	12(16.7)	49(68.1)
Research is irrelevant to my life	6(8.3)	8(11.1)	58(80.5)	6(8.3)	6(8.3)	60(83.3)
**RESEARCH DIFFICULTY**						
I find it difficult to understand the concepts of research	21(29.2)	13(18.1)	38(52.8)	15(20.8)	14(19.4)	43(59.7)
I am worried that I will make many mistakes in research	21(29.2)	16(22.2)	35(48.6)	15(20.8)	12(16.7)	45(62.5)

## Discussion

This paper presents three independent studies on research in the undergraduate medical curricula from three African institutions. Despite the independent impulse for each study, the triangulation of the findings points to some common elements. Scaria 13 presents a useful, framework for the discussion of the findings from these three studies.

The first component of his conceptual framework is initiative (exposure, experience and knowledge) or lack thereof. Both the South African and Sudanese studies described faculty inexperience and, at best, a hesitant faculty attitude towards research.

In addition, two of the studies underlined the participants' view of research being valuable in theory but the South African study added the view that research seemed out of reach while the Ugandan study emphasised the emotional response – anxiety in particular – as a result of having to do research. Anxiety can be a barrier to mastering concepts in research 22 and academic support, academic effort, and self-efficacy have been reported to be negatively correlated with research anxiety among undergraduate pharmacy students. 23 Maharajan et al. (2017) highlighted academic support as the predominant factor to reduce research anxiety. Both the South African and Sudanese studies reported hesitant faculty, which by extension, suggests low levels of academic support for students.^23^

Increasing staff engagement in undergraduate student research to develop highly motivated staff is critical to mentor highly-motivated students. 24 Staff training, protected staff time, incentives and funding to include such students in ongoing postgraduate or faculty research projects may contribute towards a more enabling and supportive undergraduate research environment.^24^ While faculty inexperience/hesitancy is a challenge that might best be overcome through supported practice, the Ugandan study confirmed a previous study 25 that showed that a simple and cheap intervention such as a short-course on research methodology can have a positive effect on the knowledge and attitudes of student participants. The inclusion of pre-designed research topics in the South African study is practical from an operational perspective but countermands personal interest or initiative.

Scaria's second component is impulse (time and competitive environment). Both the South African and Sudanese studies report on perceived time constraints from both the student participants' and faculty's perspective. The South African study also reported that student respondents thought that research was perhaps a personal, rather than a common, interest which resonates with Sca-ria's noton of a competitive environment where multiple interests compete for attention. While there is little doubt that some students will have more (or less) personal interest in research, the reality is that inclusion of research is beneficial to students.^8^

The incentive component (presentation/publication opportunities and acknowledgement) was mentioned in the South African study. The incentives related to research competence are mostly long-term and less obvious than the immediate benefits of passing modules for example. Therefore, these longer-term incentives can be more easily be deferred due to current time constraints, lack of avenues to publish student-led work or a possible lack of awareness of the benefit of research outputs on curricula vitae.

Scaria's final component is idols (mentors and supervisors). The South African and Sudanese studies confirm that research needs to be planned for in terms of timing, duration, content, place; expected output and other considerations. However, these findings expand Scaria's notion of an idol to beyond individual supervision and role modelling to a champion who drives the planning, implementation and measurement of the inclusion of research in curricula at an institutional level.

## Conclusion

The creation of competency frameworks in medical education has augmented the primacy of clinical competence with the addition of other competencies. Some of these newer competencies require research training. These three studies have used different approaches to explore this common concern and while this hampers comparison, it does underline the many ways in which we can explore and address the inclusion of research in the undergraduate medical curriculum. But the juxtaposition of these studies does provide some insights, specifically the similar challenges of providing opportunities for medical students to achieve this competency in the South African and Sudanese studies and the promising initial benefits of a short-course in Uganda. Follow-up studies are needed to test the long-term effects of inclusion of such a research short-course and to determine to what extent, if any, such a course can overcome the identified barriers.

## Limitations

The three studies were limited due to the small number of participants in each study. In addition, the different methodologies used in the South African and Sudanese studies means that the data is complementary rather than comparable. In the Ugandan study, the questionnaire did not allow exploration the results that showed a decrease between pre-test and post-test in some of the statements related to positive attitudes towards to research. The studies reviewed the perceived barriers to research. Further review of the benefits for students, faculty and universities is needed.

## Figures and Tables

**Table 4:** Attitudes towards research pre- and post-attendance of a short course in research methods (by domain)

Domain	Pre-test	Post test	P value
Research usefulness	12(11-15)	13(10-15)	0.7995
Research anxiety	25.5(21-29)	28(23-31)	0.00095
Positive attitude	13(10-15)	12(9-14.5)	0.2794
Research relevance	13(12-15)	13(12-15)	0.8445
Research difficulty	6(5-8)	7.5(6-8)	0.0705
